# Intraoperative Digital Analysis of Ablation Margins (DAAM) by Fluorescent Confocal Microscopy to Improve Partial Prostate Gland Cryoablation Outcomes

**DOI:** 10.3390/cancers13174382

**Published:** 2021-08-30

**Authors:** Oscar Selvaggio, Ugo Giovanni Falagario, Salvatore Mariano Bruno, Marco Recchia, Maria Chiara Sighinolfi, Francesca Sanguedolce, Paola Milillo, Luca Macarini, Ardeshir R. Rastinehad, Rafael Sanchez-Salas, Eric Barret, Franco Lugnani, Bernardo Rocco, Luigi Cormio, Giuseppe Carrieri

**Affiliations:** 1 Department of Urology and Organ Transplantation, University of Foggia, 71122 Foggia, Italy; oscarsel@libero.it (O.S.); mariano.bruno91@gmail.com (S.M.B.); marco.recchia291292@gmail.com (M.R.); luigi.cormio@unifg.it (L.C.); giuseppe.carrieri@unifg.it (G.C.); 2 Department of Urology, Azienda Ospedaliero-Universitaria di Modena, 41121 Modena, Italy; sighinolfic@yahoo.com; 3 Department of Urology, ASST Santi Paolo e Carlo Dipartimento di Scienze della Salute, Università degli Studi di Milano, 20122 Milano, Italy; bernardo.rocco@gmail.com; 4 Department of Pathology, University of Foggia, 71122 Foggia, Italy; fradolce@hotmail.com; 5 Department of Radiology, University of Foggia, 71122 Foggia, Italy; paola.milillo@yahoo.com (P.M.); luca.macarini@unifg.it (L.M.); 6 Department of Urology, Lenox Hill Urology, Northwell Health System, New York, NY 10075, USA; arastine@northwell.edu; 7 Urology Department, Institut Mutualiste Montsouris, 75014 Paris, France; rafael.sanchez-salas@imm.fr (R.S.-S.); Eric.Barret@imm.fr (E.B.); 8 Hippocrates D.O.O. Center, 6215 Divaca, Slovenia; info@lugnani.com; 9 Department of Urology, Bonomo Teaching Hospital, 76123 Andria, Italy

**Keywords:** prostate cancer, fluorescence confocal microscopy, prostate biopsy, ablation margins, focal therapy

## Abstract

**Simple Summary:**

This study tested the feasibility and reliability of a novel digital microscopy technique in assessing ablation margins during partial prostate gland cryoablation. Though preliminary, findings suggest that this novel technique may increase the efficacy of focal treatments, by reducing the risk of untreated prostate cancer areas not visible to an MRI, as well as safety, by more precisely sparing uninvolved areas and surrounding structures.

**Abstract:**

Partial gland cryoablation (PGC) aims at destroying prostate cancer (PCa) foci while sparing the unaffected prostate tissue and the functionally relevant structures around the prostate. Magnetic Resonance Imaging (MRI) has boosted PGC, but available evidence suggests that ablation margins may be positive due to MRI-invisible lesions. This study aimed at determining the potential role of intraoperative digital analysis of ablation margins (DAAM) by fluoresce confocal microscopy (FCM) of biopsy cores taken during prostate PGC. Ten patients with low to intermediate risk PCa scheduled for PGC were enrolled. After cryo-needles placement, 76 biopsy cores were taken from the ablation margins and stained by the urologist for FCM analysis. Digital images were sent for “real-time” pathology review. DAAM, always completed within the frame of PGC treatment (median time 25 min), pointed out PCa in 1/10 cores taken from 1 patient, thus prompting placement of another cryo-needle to treat this area. Standard HE evaluation confirmed 75 cores to be cancer-free while displayed a GG 4 PCa in 7% of the core positive at FCM. Our data point out that IDAAM is feasible and reliable, thus representing a potentially useful tool to reduce the risk of missing areas of PCa during PGC.

## 1. Introduction

EAU guidelines mention partial gland cryoablation (PGC) as a minimally invasive investigational option for the management of organ-confined prostate cancer (PCa) [1]. One of the technical challenges during PGC is to deploy multiple cryo-needles to form an ablation zone sufficiently covering the target volume while sparing surrounding critical structures. Historically, physicians relied on digital rectal examination (DRE) and template prostate biopsy results to plan the cryo-needles placement. The multifocal nature of PCa and the suboptimal prostate sampling obtained using template biopsies, however, limited the widespread use of PGC and supported treatment of the entire gland or at least half of it (hemigland ablation) to achieve wide safety margins. Even so, up to 15% of patients having undergone hemigland cryoablation experienced treatment failure at 5 years of follow-up [2]. The availability of multiparametric Magnetic Resonance Imaging (MRI) has boosted PGC. Indeed, the combination of MRI and target biopsy have been proved to be superior to template prostate biopsy alone in identifying lobes with significant PCa for the application of hemi-ablative focal therapies [3].

Recent studies reported encouraging mid-term results in cohorts of patients undergoing MRI-guided focal therapies [4,5]. Still, MRI reader experience [6], MRI-invisible lesions, and targeting errors in placing the cryo-needles [7] can lead to positive ablation margins impacting PGC outcomes.

Recently, a novel technology has shown promise for the real-time microscopic evaluation of prostate tissue [8]. Fluorescent confocal microscopy (FCM) allows the immediate acquisition of digital images in a hematoxylin-eosin (HE)-like fashion without conventional processing and its attendant time and resource requirements. In the preliminary studies, FCM provided an excellent discrimination performance compared to HE for prostate biopsy cores and peri-prostatic tissue evaluation [9,10].

This pilot study aimed at determining feasibility and reliability of intraoperative FCM in assessing ablation margins during PGC.

## 2. Materials and Methods

### 2.1. Study Population

Following ethics committee approval (University of Foggia, approval number 143/CE/2020), ten patients with clinically localized low to intermediate risk PCa (PSA ≤ 20.0 ng/mL, Gleason Group (GG) ≤ 3) scheduled for PGC between September and November 2020 were enrolled in this prospective study evaluating intraoperative digital analysis of ablation margins (DAAM) by FCM. Patients with high-risk PCa (PSA > 20.0 ng/mL, GG > 3) and those who received any prior treatments for malign and benign prostatic disease were excluded.

All patients meeting inclusion and exclusion criteria signed an informed consent form.

### 2.2. Multiparametric Magnetic Resonance Imaging and Prostate Biopsy Technique

A prebiopsy mpMRI was performed and interpreted by a single dedicated radiologist (7 years’ experience in prostate MRI) according to PIRADS V2.1 recommendation [11]. Specifically, the mpMRI protocol consisted of: (a) Turbo-Spin-Echo (TSE) T2-weighed imaging in axial, coronal, and sagittal planes (repetition time (TR) 5300, echo time (TE) 150 ms, slice thickness 3 mm, field of view (FOV) 180 × 180, number of signals averaged (NSA 8); (b) TSE T1-weighed imaging in the axial plane (TR/TE 400–650/12 ms, thickness 3 mm, FOV 180×180, NSA 3); (c) Diffusion-weighted imaging sequence (DWI) in the axial plane (TR/TE 3481/92 ms, slice thickness 3 mm, FOV 180 × 220, NSA 4, b-values 0–500–1000–1500/2000 sec/mm^2^); (d) Dynamic contrast enhanced prostate MRI was performed using a T1-weighted high-resolution isotropic volume examination (THRIVE) on the axial plane (TR/TE 4.5/2.2 ms, slice thickness 3 mm, FOV 184 × 220, NSA 1) following injection of 0.1 mL/kg of gadobutrol followed by 20 mL of saline solution using an automatic injector at a rate of 2 mL/s. All patients underwent prostate biopsy at our institution with 2 to 4 target cores to any MRI suspicious lesion in addition to our 18 cores systematic template [12]. An Electromagnetically Tracked MRI/US Fusion system (Navigo, UC-Care, Tampa, FL, USA) was used to performed target sampling.

### 2.3. Prostate Gland Cryoablation with Intraoperative Digital Analysis of Ablation Margins (DAAM)

**Figure 1:**
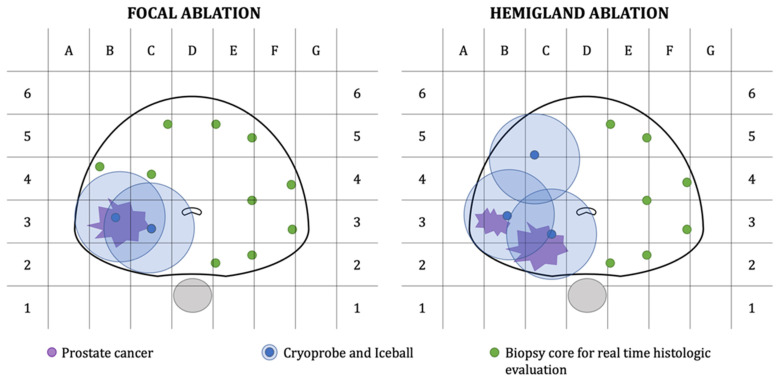
Graph representation of treatment plan and biopsy core template for patients undergoing focal or hemigland cryoablation.

Based on mpMRI findings and biopsy results, two to four 2.4 mm cryo-needles were inserted transperineally using a template grid and with the help of an Electromagnetically Tracked MRI/US Fusion system (Navigo, UC-Care, Tampa, FL, USA). Prostate Cryoablation was performed using an argon/helium gas-based system (Endocare, HeathTonics Inc., Austin, TX, USA). After the creation of the ice balls, 7 to 10 biopsy cores, depending on prostate volume, were taken transperineally from ablation margins and untreated areas of the prostate (Figure 1). In order to avoid potential tissue damage, cores were taken outside ice balls. A single surgeon performed PGC and intraoperative prostate biopsies (OS).

The cores were prepared for FMC (VivaScope^®^ 2500 M-G4, VivaScope GmbH, Munich, Germany) in the operating room by a urologist (UF) following the manufacturer guidelines. A glass slide is loaded with three biopsy cores and then scanned within a maximum time of 2 min. Sample preparation and image acquisition do not require special training. Samples are first cleaned using 70% Ethanol solution and then colored using Acridine Orange (1/50 solution in distilled water for 20–30 seconds). There are no specific requirements for the cores (e.g., length, diameter, thickness, integrity); however, to reduce acquisition time, care needs to be taken to place each biopsy core parallel or perpendicular to the slide axis. The FCM device combines two different lasers that enable tissue examination according to reflectance (785 nm) and fluorescence (488 nm) modalities. Images are rendered by the microscope software as pseudo-Hematoxylin-eosin (HE) images, relying on the combination of two images acquired at each wavelength. Specifically, nuclei appear purple, and collagen and cytoplasm appear pink.

The obtained digital FCM images were sent for “real-time” pathology review to a dedicated uropathologist (FS). Prostate Cryoablation was completed with no adjustments in patients with negative ablation margins. Conversely, if one or more cores were positive at FCM evaluation, the surgical plan was modified in order to ensure treatment of all cancer areas.

### 2.4. Final Pathology Examination

Biopsy cores were then fixed, stained using standard hematoxylin & eosin (HE), and sent to the pathology department for final diagnosis. A second dedicated uropathologist, blinded to clinical information and digital biopsy results, reported all PB specimens according to the 2019 ISUP recommendations [13] and diagnostic criteria for high-grade prostatic intraepithelial neoplasia and atypical small acinar proliferation of prostate [14]. ISUP Gleason-grade groups (ISUP GG) were reported per each core.

### 2.5. Study Endpoints and Statistical Analysis

The primary endpoint of the present study was time for FCM diagnosis. The secondary endpoint was the efficacy of FCM as measured by the agreement between digital biopsies and standard HE evaluation in the assessment of PCa. Surgical and intraoperative biopsy complications were reported using the Clavien-Dindo Classification [15]. Finally, PSA values at three months after therapy were used to compute the percentage of PSA reduction (derived from the ratio between PSA at three months and preoperative PSA) [16].

Descriptive statistics were performed using Stata 14 (StataCorp LP, College Station, TX, USA).

## 3. Results

**Figure 2:**
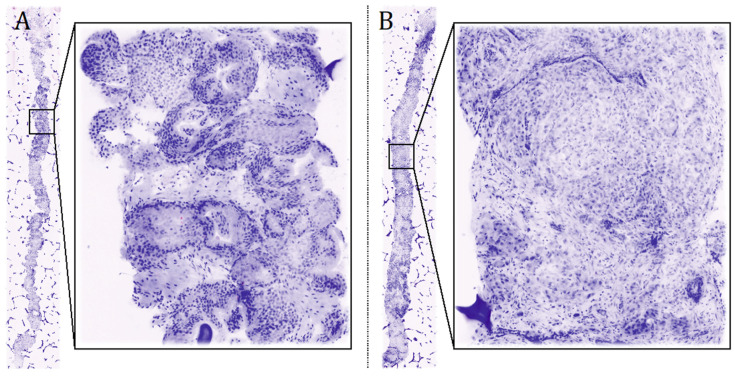
Digital Biopsy images. High-resolution pictures allow zooming into focal areas to better evaluate the architecture of the prostatic gland. (**A**) Negative core and zoom into normal gland architecture. (**B**) Positive core, Gleason Grade Group 4, pattern 4.

Descriptive characteristics of the study population are listed in Table 1. Four patients with biopsy-proven MRI-visible GG1-2 PCa were treated with focal Cryoablation. The remaining patients with unilateral non-MRI-visible PCa underwent prostate hemiablation according to the SPARED guidelines [17]. In total, 76 cores were taken and analyzed using FCM (Figure 2). The median time for FCM diagnosis was 25 (IQR: 25, 27) min, with DAAM being always completed before the conclusion of the two treatment cycles. DAAM was negative in 9 patients, whereas in one patient with a preoperative diagnosis of low volume (two cores) GG 2 PCa, it pointed out PCa in 1 of 10 cores from the ablation margin area (left medial posterior core). A third cryo-needle was then placed to treat this positive core. The post-operative stay was uneventful, and all patients were discharged on post-operative day 1 with a urethral catheter removed on post-operative day 7. The median PSA drop at three months was 5.7 (IQR: 4.7, 6.6) ng/mL. The median percentage of PSA reduction was 79.0 (78.0, 85.0).

Standard HE evaluation confirmed 75 cores to be cancer-free while displayed a GG 4 PCa in 7% of the core positive at FCM.

## 4. Discussion

To our knowledge, this is the first study testing the potential role of intraoperative digital analysis of ablation margins (DAAM) by fluorescence confocal microscopy during prostate PGC. This novel technique holds promises to revolutionize PCa diagnosis and treatment by potentially replacing traditional frozen sections.

Other optical technologies allowing real-time microscopic evaluation of cancer tissue have been recently described and can be divided into two main groups. Conventional confocal microscopy is the most cost-effective option. UV light-emitting diode and other lasers with different wavelengths have been tested for real-time microscopic examination of fresh tissue taken from different cancers (mainly skin, prostate, and breast cancer) [18,19,20,21]. The technical process for image acquisition is the same; all light sources provided optimal quality images; however, comparative studies are lacking.

On the other hand, multiphoton microscopy involves an ultrafast (typically femtosecond pulse duration) laser source to achieve the extremely high photon density at the focal plane needed to excite two-photon absorption-based fluorescence [22,23]. This technique achieves a considerably higher imaging depth (using near-infrared wavelengths) with comparable image resolution, but it is limited by longer acquisition times and much higher costs of the microscope.

While the best technique for image acquisition is still a matter of debate, the relevance of the intraoperative analysis of prostatic tissue was clearly pointed out by the NeuroSAFE dissection technique, which was developed to maximize the preservation of periprostatic tissue during nerve-sparing radical prostatectomies [24]. While the patient is still under general anesthesia, the posterolateral aspect of the prostate is prepared using a cryostat, stained with HE, and analyzed under the microscope by a dedicated pathologist on-site. If persisting malignant glands are noted, a secondary resection of the ipsilateral bundle is performed. Though promising, it required a dedicated setup to obtain a diagnosis in a reasonable time [25].

To overcome these limitations, Rocco et al. proposed using FCM for real-time assessment of surgical margins during radical prostatectomy, reporting excellent results [10]. Indeed, the pathologist provided results from remote in less than 25 min, and there was perfect agreement between FCM and subsequent HE findings.

FCM may be even more crucial in the context of focal therapies. Indeed, frozen sections are not a viable option, and FCM is the fastest technique allowing the preservation of biopsy cores for further HE staining, immunohistochemistry/genomic studies.

The two studies tested the feasibility and diagnostic accuracy of FCM onto prostate biopsy cores rather than surgical margins. Rocco et al. obtained digital images of biopsy cores in 54 patients; perfect agreement between FCM and HE diagnosis was obtained for 95.1% of the 427 tested cores [9]. Similarly, Marenco et al. tested by FCM 182 MRI-targeted biopsy cores obtained in 57 biopsy-naive patients. The median time for FCM processing and analysis was 5 minutes; the positive and negative predictive values were 85% and 95%, respectively [26].

The present study confirmed the perfect agreement between FCM and standard HE findings, with FCM not affecting at all the quality of HE images in all 76 taken cores.

Our findings open novel perspectives on focal therapies since real-time detection of PCa in the ablation margins is likely to dramatically reduce the risk of undertreatment and, therefore, of disease recurrence. All patients included in the present study had a reduction of PSA > 75% at three months of follow-up. Even if the percentage of PSA reduction represents only a surrogate of the oncological outcome, Stabile et al. recently showed it is inversely associated with disease recurrence and the need for additional treatment, thus potentially useful as a proxy of treatment efficacy [16].

The small sample size and short-term follow-up are obvious limitations of this pilot study which, however, provides solid grounds for further evaluation of DAAM in the setting of focal PCa treatments.

## 5. Conclusions

Intraoperative DAAM by FCM proved to be feasible and reliable. This strategy potentially allows to reduce disease recurrence by extending treatment to previously unsuspected areas of PCa as well as to reduce functional complications by limiting treatment to the affected areas while sparing surrounding structures.

## Figures and Tables

**Table 1:** Descriptive characteristics of the study population.

	Study Population (*N* = 10)
Age, years	70 (68, 73)
PSA, ng/mL	6.9 (6.2, 8.9)
DRE, n (%)	
Negative	4 (40.0%)
Positive	6 (60.0%)
PIRADS, n (%)	
2	2 (20.0%)
3	2 (20.0%)
4	4 (40.0%)
5	2 (20.0%)
Prostate Volume, cc	41.5 (40.0, 50.0)
Positive cores	3.5 (3.0, 5.0)
Bx GG, *n* (%)	
1	4 (40.0%)
2	4 (40.0%)
3	2 (20.0%)
EAU risk, *n* (%)	
Low risk	3 (30.0%)
Intermediate Risk	7 (70.0%)
Treatment, *n* (%)	
Focal ablation	4 (40.0%)
Hemigland Ablation	6 (60.0%)
N of Probes	3.5 (2.0, 4.0)
Intraoperative cores, *n* (%)	
7	8 (80.0%)
10	2 (20.0%)
Time to FCM diagnosis	25 (25, 27)
3-month PSA, ng/mL	1.4 (1.0, 1.6)
PSA drop, ng/mL	5.7 (4.7, 6.6)
Percentage of PSA reduction	79.0 (78.0, 85.0)

## Data Availability

Data supporting reported results can be obtained upon reasonable request to the corresponding author.
